# Growth inhibition of HeLa cell by internalization of *Mycobacterium bovis *Bacillus Calmette-Guérin (BCG) Tokyo

**DOI:** 10.1186/1475-2867-9-30

**Published:** 2009-12-02

**Authors:** Akira Kitamura, Sohkichi Mastumoto, Izumi Asahina

**Affiliations:** 1 Division of Oral and Maxillofacial Surgical Reconstruction and Functional Restoration, Department of Developmental and Reconstructive Medicine, Graduate School of Biomedical Sciences, Nagasaki University, Sakamoto, Nagasaki 852-8588, Japan; 2 Department of Host Defense, Osaka City University. Graduate School of Medicine, Asahi-machi, Abeno-ku, Osaka 545-8585, Japan

## Abstract

**Background:**

Intravesical BCG immunotherapy is effective for preventing recurrence and progression in none muscle-invasive bladder cancer but the dosing schedule and duration of treatment remain empirical. The mechanisms by which intravesical BCG treatment mediates antitumor activity are currently poorly understood.

**Results:**

HeLa cell infected with *Mycobacterium bovis *Bacillus Calmette-Guérin(BCG) Tokyo which were different multiplicity of infection(MOI). Proliferation of HeLa cell reduced in a dose-dependent manner by live BCG. The cytoplasm of the HeLa cell showed variety lysosomal stages by internalized and interacted BCG.

**Conclusion:**

Proliferated Live BCG secreted the protein and depressed the growth of tumor. The possibility for clinical introduction of BCG therapy for carcinoma reported with review of literature.

## Background

Intravesical BCG treatment has been demonstrated to be an effective therapy for superficial transitional cell carcinoma of the bladder though the mechanism of antitumor effect still remained unclear. We studied to whether the BCG depressed the growth of malignant tumor cell or not. The proliferation of HeLa cells were inhibited by dose dependent manner infected by live BCG. HeLa cell infected by live and dead *Mycobacterium bovis *Bacillus Calmette-Guérin(BCG) Tokyo revealed variety stage of lysosome and BCG in the cytoplasm of HeLa cell. The internalization of live BCG into the HeLa cells did not blocked by heparin and cytochalasin B. The internalized live BCG secreted the secreted-protein and depressed the growth of tumor cell. Live BCG inhibited the growth of tumor cell by internalized and then the secreted protein in cytoplasm of HeLa cells suggested the possibility of new cancer therapy made of BCG combined with the drug delivery system(DDS).

## Results

**Figure 1:**
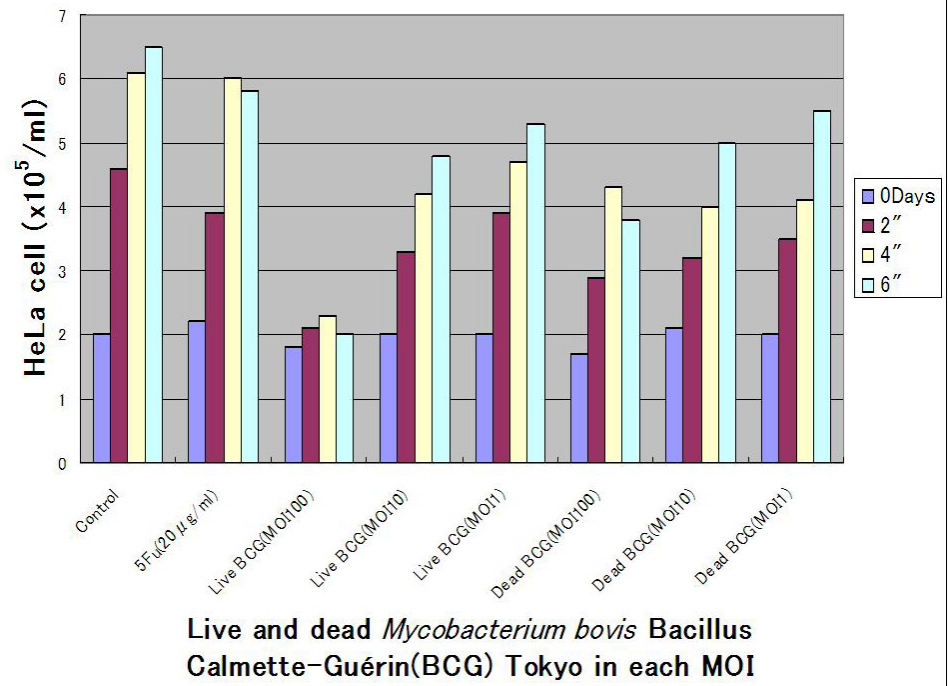
**Growth of HeLa cells were depressed by live and dead *Mycobacterium bovis *Bacillus Calmette-Guérin(BCG) Tokyo in each multiplicity of infection(MOI)**. The growth of HeLa cells were inhibited in dose dependent manner cultured by live BCG. The proliferation of HeLa cell was not inhibited when the MOI was 1.

**Figure 2:**
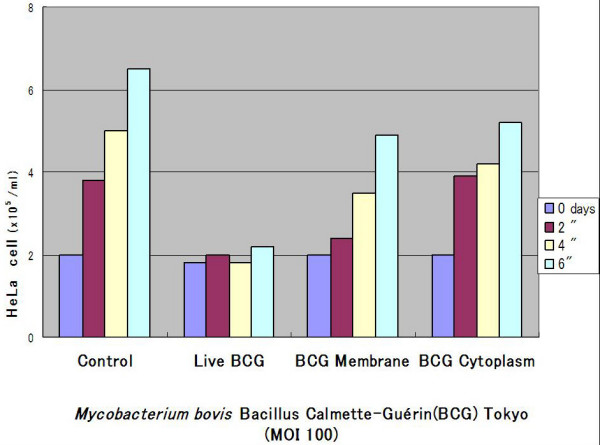
**Growth depression of HeLa cell by different BCG component(MOI 100)**. Live BCG depressed the tumor growth than that of the membrane(0.04 μg/ml, DW) and cytoplasm(0.02 μg/ml, DW) fraction each dosage were equivalent of MOI 100.

The growth of HeLa cell was inhibited by dose dependent manner cultured by live *Mycobacterium bovis *Bacillus Calmette-Guérin(BCG) Tokyo (Figure 1). The proliferation of HeLa cell was not inhibited when the MOI was 1. Live BCG indicated more depressed the growth of HeLa cell than the each membrane(0.04 μg/ml, dry weight) or cytoplasm(0.02 μg/ml, dry weight) fraction of the BCG [1] whose dosage were equivalent of MOI 100 (Figure 2).

**Figure 3:**
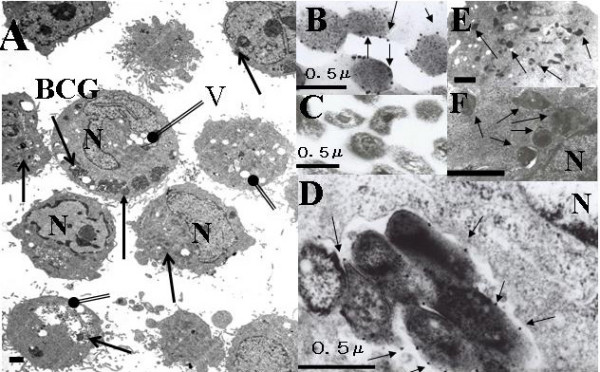
**A; HeLa cell and live BCG were co-cultured after 2 days**. There were vacuole(V) and BCG(↑) in the cytoplasm of HeLa cell. B, D; Protein A gold(↑) attached on the cell wall of BCG in the HeLa cell co-cultured with live BCG after two days by immunoelectron microscope (anti MPB70 polyclonal antibody). C; Dead BCG did not reacted at the cell wall. Cytochalasin B (E, 100 μg/ml) or Heparin (F, 0.001 U/ml) was added in each culture well of HeLa cell before infection by live BCG also showed the internalized BCG(↑) after 24 hours. N; Nucleus

**Figure 4:**
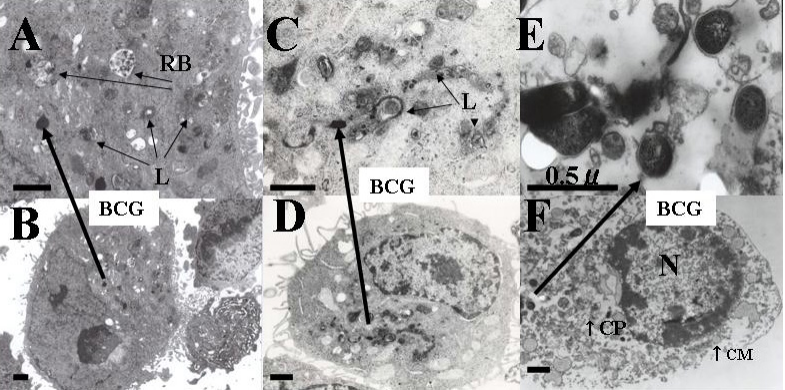
**A, B; HeLa cells were infected with live BCG after 1 hour and showed BCG(↑), residual body(RB) and lysosome(L) in the cytoplasm**. Different stages of the lysosomal activity were induced by internalized BCG. C, D; HeLa cells were co-cultured with dead BCG(↑) which were also internalized after 1 hour and showed the lysosome and vacuole. E, F; HeLa cells co-cultured with live BCG after four days. HeLa cell showed lack of cell membrane(↑CM), lost of cytoplasm(↑CP), nuclear degeneration(N) and BCG(↑). The shape of BCG was keeping in the HeLa cell.

Two days after cultured with live BCG (MOI 100), HeLa cell showed vacuole and BCG in the cytoplasm (Figure 3A). Even one hour after cultured with live BCG, the cytoplasm of HeLa cell also revealed lysosome, residual body and BCG (Figure 4A). There were several kinds of lysosome which indicated phagocytosis caused from internalized BCG (Figure 4A, C). The myelin-like multilamellar structure was also recognized in the cytoplasm of HeLa cell by dead BCG one day after incubated [2] (Figure 4C). Internalized live or dead (Figure 4A, C) BCG induced the lysosomal activity of the HeLa cell. Four days after the infected HeLa cell by live BCG showed the necrosis in which the BCG kept its shape (Figure 4E). The internalization of live BCG into the HeLa cells was found in their cytoplasm with cytochalasin B [3] (100 μg/ml, Figure 3E) or heparin [4] (0.001 U/ml.Figure 3F) was added into each well before co-culture with HeLa cell respectively. Immunoelectron microscope checked using polyclonal antibodies of the MPB70 (secreted protein, α-antigen) [5-7] and revealed the protein A gold reacted around the cell wall of live BCG. (Figure 3B, D)

## Discussion

Intravesical Bacillus Calmette-Guérin(BCG) therapy has been effective in delaying or preventing recurrence and progression for transitional cell carcinoma of bladder although its outcome is still unpredictable [8]. The report suggested that BCG interacted with tumor cells or internalized into them, and yet the role of cellular attachment has been un-established. Therefore we initiate studied using the TEM to better define for the interaction of HeLa cells with BCG and leading to the hypothesis that live BCG induced anti-tumor activity in the tumor cells. The growth inhibition of the HeLa cell was more distinct by live BCG to compare dead BCG, the cytoplasm or membrane fraction of BCG [1]. Those indicated live BCG invaded and proliferated in the tumor cells. Since BCG infection inhibits the proliferation and differentiation of HeLa cells, question arises as to the mechanism whether inducing the bacteriological function from inside or outside. Several bacterial components have already been reported the affected proliferation by infection [9]. The possibility remains that poor nutrition of the cells caused by intracellular proliferation of BCG as well as the BCG stimulus on the cell surface receptor may also be involved in the suppression of cellular proliferation and differentiation [2].

Infection and growth of live BCG in the host cell and released BCG-related cytokine were estimated as the reasons for the depression of the tumor cells. MPB70 [7] (α antigen) known to be an immunogenic mycobacterial protein secreted in large amounts from culture filtrated of *Mycobacterium bovis *Bacillus Calmette-Guérin(BCG) Tokyo. This protein is thought to be crucial for binding phagocytic cell having fibronectin receptors and this function might be a direct effect of BCG immunotherapy[10,11]. BCG is thought to bind to the bladder wall via interaction between the bacterial antigen complex and fibronectin [12]. Similar observations with fibronectin attachment protein(FAP) demonstrated a Type I response inducing IL12 and IFNγ production in normal human peripheral blood lymphocytes. These data suggest that a Type I response is required for antitumor activity by BCG [13].

Antitumor effects of BCG against superficial urinary bladder cancer were known to be strong when BCG is directly infused into the bladder, but its immunological mechanisms are poorly understood [14]. The internalization was inhibited by cytochalasin B(200 μg/ml)[3]; conditions known to inhibit phagocytosis [15]. Heparin (1.25 U/Kg) also induced the aggregation of the local expression of fibronectin and sequentially lessen FN-mediated BCG attachment to bladder wall [4]. But cell membrane-expressed fibronectin did not seem to be crucially involved in the internalization of BCG by transitional bladder cancer. A correlation between cellular fibronectin expression and the ability of transitional cell carcinoma to internalize BCG may be considered as a fortuitous coincidence [16]. Our data showed the internalization of live BCG into the HeLa cells were not blocked by heparin or cytochalasin B. These phenomena suggested another possible reaction between the membrane of the HeLa cell and BCG. Further experiments will be necessary to clarify the biological relevant related between the internalization and phagocytosis or autophagy which is associated with the role of mycobacterial infection and intracellular killing of the cell [17]. Autophagy, the process in which cellular organelles are targeted for degradation in lysosome, represents another potential tumor resistance mechanism and further adding to the complexity of cell death pathways when tumor cells are exposed to various agents [18,19]. IFN-γ induction of autophagy has not been previously reported in immune or phagocyte cells but has been observed in HeLa cells [2,20]. We reported that mycobacterial infection induces the Th1-type immune response lead on the immunological environment rich in IFN-α, which is a suppressive mediator of the Th2-type immune reaction [21]. Th1-stimulating cytokines played an important role in BCG-induced macrophage cytotoxicity and that combination of BCG with selected Th1-stimulating cytokines, either supplemented or expressed by BCG, may enhance the effect of BCG in the treatment of bladder cancer patients [22]. Early stages of BCG infection into osteoblastic-like cell (MC3T3-E1) secreted IL-6 and then depressed the proliferation of host [23]. The findings that live BCG infected and internalized in the HeLa cells as shown here, lysosomal activity is an important connection between immune mediator and associated intracellular depression of the host cells. These data suggested that live BCG invaded and proliferated in the cell then released the BCG-related protein have direct effect to inhibit the growth of the host.

The novel method such as using target would allow further improve the bacteria to satisfy a variety of requirements for clinical use. These therapies elicit active immune response against the tumor so that they kill off primary as well as metastases [24]. Successful cancer therapy required close contact between BCG and tumor cells, a host capable of developing and expressing delayed hypersensitivity type reactions to mycobacterial antigens, limited tumor size and an adequate number of viable BCG [25]. It is also necessary to establish the effective drug delivery system(DDS) to be internalized into malignant cell in vivo treatment [26]. The difference between the live and dead BCG which internalized in the cytoplasm of HeLa cell is the existence of secreted protein or not. BCG interacted with tumor cells and were internalized into them suggested future development of anti-tumor agents made from bacterial cell wall [27]or secreted protein [28].

## Conclusion

Live BCG depressed the growth of the HeLa cell by dose dependent manner.

Live BCG internalized and secreted protein in the host cell suggested the depression of tumor cell.

## Methods

### HeLa cell

HeLa cell (1 × 10^5 ^cells/ml) were maintained in minimal essential medium (MEM, Gibco BRL, Tokyo Japan) supplemented with 10% fetal bovine serum and 100,000 U/1 penicillin at 37°C in humidified atmosphere with 5% CO_2_. The cultured medium was replaced every 3 days. Cells were rinsed 2 times with phosphates-buffered saline (PBS; 137 mM NaCl, 2.7 mM KCl, 8.1 mM NaH_2_PO_4_) before addition of fresh medium.

### Bacillus Calmette-Guérin(BCG) [7,1,29]

*Mycobacterium bovis *Bacillus Calmette-Guérin(BCG) Tokyo was cultured in Middle brock 7H9 broth (Difco Laboratories, Detroit, MI, USA) supplemented with 10% Albumin-dextrose-catalase(ADC; Difco laboratories) enrichment and 0.1% Tween 80. The cells(approximate 8 × 10^10 ^cells/ml) were harvested with shaking at 37°C until 0.8 of an optical density(OD) at 590 nm. It was centrifuged and the pelletized cells re-suspended with MEM were divided and used for experiments. Dead BCG dosage (1 × 10^7 ^cells/ml) as MOI 100 was prepared and treated by an autoclave (121°C, 10 min).

### Fraction of BCG membrane and cytoplasm [1]

BCG(Tokyo) was sedimented (3,000 × g, 10 min, 4°C), suspended in TMNSH buffer, and lysed by sonication in a Bioruptor UCD-200T sonicator (Toso, Tokyo, Japan). The cell lysate was centrifuged at 10,000 × g for 10 min at 4°C twice. The supernatant was then centrifuged at 30,000 × g for 30 min at 4°C. The pellet was used as the membrane fraction.

The supernatant solution was centrifuged under the same conditions, and the supernatant thus obtained was then centrifuged at 105,000 × g for two hours at 4°C. The pellet was used as the cytoplasm fraction.

The pellets obtained in each step were suspended in TMNSH buffer. Freeze drying membrane and cytoplasm fraction were prepared 0.04 μg/ml and 0.02 μg/ml respectively for the experiment as MOI 100 and keeping at the department of oral bacteriology Nagasaki University. BCG also had been cultured in the same department.

### Polyclonal antibody [7]of MPB70 (secreted protein, α antigen) [5,6,10]

Purified MPB70 was provided by Dr. Nagai. BALB/c mice at 7-10 weeks of age were immunized intravenously with MPB 70 (10 μg diluted 200 ul of PBS) which was served as the most abundant protein in the culture filtrate from BCG(Tokyo). After 30 days, same amount of MPB 70 was injected intraperitoneally to boost the immune response. After 1 week later, sera were collected from the eye vein of immunized mice and pooled at -80°C until use. Animal had been keeping at animal center of Nagasaki Univ.

### HeLa and BCG cells co-culture

Two millimeter of HeLa cells (1 × 10^5^/ml) were inoculated in 24-well plates and cultured for 1 days. Then BCG at various doses and type were added to the wells. After 72 hours fresh MEM exchanged one ml.

Different BCG dosage even, ten and hundred times of multiplicity of infection (MOI) BCG were prepared and cultured with HeLa cell.

Growth inhibition of HeLa cell checked by its fraction of membrane (0.04 μg/ml, Dry weight) and cytoplasm (0.02 μg/ml, Dry weight) were also prepared as equivalent dosage of MOI 100 and added in the medium of HeLa cells.

Internalization of the BCG checked using each cytochalasin B (100 μg/ml, Wako pure C.I. Japan) or heparin sodium (0.001 U/ml, OSTUKA Pharm. Japan) was added into the well before co-cultured with HeLa cell respectively.

### Cell count

Every other day during the incubation period with the HeLa and BCG, MEM changed to the usual saline solution and treated 2% tripsin treated for 5 minutes. HeLa cells were mounted on the erythrocytometer after 0.3% trypanblue stain. The number of cells expressed as the mean for three times.

### Transmission electron microscope (TEM) [28]

The HeLa cell washed in the normal saline solution and centrifuged. The cells were fixed in solution of 2% glutaraldehyde in 0.1 M phosphate buffer solution, pH 7.3 for 2 hours and 1% osmium tetraoxide for 2 hours. After two times washed PBS and water the cells were dehydrated with increasing concentrations of ethanol; and gradually infiltrated with Epon 812. Before inspection by TEM the trimmed bloc of epon was orientated and stained with toluidine blue for light microscopy orientation. The ultrathin section with silver to gold interference color were picked up in a nickel grid and stained with uranyl acetate and lead nitrate in the usual manner.

For Immuno-TEM HeLa cell fixed 1% paraformaldehyde at 4°C for one hour. Then dehydrated in ethanol and embedded by LR white(Okenshoji, Japan) for 2 days at -20°C. The same interference color picked up on the collodion coated mesh (Nissinn EM, Japan) specimens were reacted with 2% hydrogen peroxide for 30 minutes twice and blocked bovine serum for 30 minutes. They were reacted with hundred times diluted anti-sera (polyclonal Aanti-MPB70) for 12 hours at 8°C and protein A gold (15 nm, FUNAKOSI, Japan) for three hours at room temperature in moisture chamber and then double stained for 5 minutes each. The specimens were examined in a H800 electron microscope (Hitachi, Japan) operating at 75 kV.

## Competing interests

The authors declare that they have no competing interests.

## Authors' contributions

AK counted the HeLa cells, carried out the morphologic study of TEM and immuno-TEM, drafted the manuscript and designed this experiment. SM cultured the BCG, prepared MPB70 and BCG fraction of membrane and cytoplasm and made the polyclonal antibody of MPB70 (secreted protein, α-antigen). IA carried out the co-culture of BCG and HeLa cell. All authors read and approved the final manuscript.
